# Epidemiology and outcomes in patients with anemia of CKD not on dialysis from a large US healthcare system database: a retrospective observational study

**DOI:** 10.1186/s12882-022-02778-8

**Published:** 2022-04-30

**Authors:** Lois Lamerato, Glen James, Heleen van Haalen, Katarina Hedman, James A. Sloand, Amy Tang, Eric T. Wittbrodt, Jerry Yee

**Affiliations:** 1 Department of Public Health Sciences, Henry Ford Health System, 1 Ford Place – 3E, Detroit, MI 48202 USA; 2 Cardiovascular, Renal, Metabolism Epidemiology, BioPharmaceuticals Medical, AstraZeneca, Cambridge, UK; 3 Integrated Evidence Generation & Business Innovation, Bayer PLC, Reading, UK; 4 Global Health Economics and Payer Evidence, BioPharmaceuticals Medical, AstraZeneca, Gothenburg, Sweden; 5 Late Cardiovascular, Renal, Metabolism, BioPharmaceuticals R&D, AstraZeneca, Gothenburg, Sweden; 6 Present affiliation: Division of Kidney Diseases & Hypertension, the George Washington University, Washington, DC USA; 7 Global Medical Affairs, Cardiovascular, Renal and Metabolism, BioPharmaceuticals Medical, AstraZeneca, Gaithersburg, MD USA; 8 Cardiovascular, Renal, Metabolism Epidemiology, BioPharmaceuticals Medical, AstraZeneca, Gaithersburg, MD USA; 9 Division of Nephrology and Hypertension, Henry Ford Hospital, Detroit, MI USA

**Keywords:** Anemia, Chronic kidney disease, Real-world evidence

## Abstract

**Background:**

Optimal management of anemia of chronic kidney disease (CKD) remains controversial. This retrospective study aimed to describe the epidemiology and selected clinical outcomes of anemia in patients with CKD in the US.

**Methods:**

Data were extracted from Henry Ford Health System databases. Adults with stages 3a–5 CKD not on dialysis (estimated glomerular filtration rate < 60 mL/min/1.73m^2^) between January 1, 2013 and December 31, 2017 were identified. Patients on renal replacement therapy or with active cancer or bleeding were excluded. Patients were followed for ≥12 months until December 31, 2018. Outcomes included incidence rates per 100 person-years (PY) of anemia (hemoglobin < 10 g/dL), renal and major adverse cardiovascular events, and of bleeding and hospitalization outcomes. Adjusted Cox proportional hazards models identified factors associated with outcomes after 1 and 5 years.

**Results:**

Among the study cohort (*N* = 50,701), prevalence of anemia at baseline was 23.0%. Treatments used by these patients included erythropoiesis-stimulating agents (4.1%), iron replacement (24.2%), and red blood cell transfusions (11.0%). Anemia incidence rates per 100 PY in patients without baseline anemia were 7.4 and 9.7 after 1 and 5 years, respectively. Baseline anemia was associated with increased risk of renal and major cardiovascular events, hospitalizations (all-cause and for bleeding), and transfusion requirements. Increasing CKD stage was associated with increased risk of incident anemia, renal and major adverse cardiovascular events, and hospitalizations.

**Conclusions:**

Anemia was a prevalent condition associated with adverse renal, cardiovascular, and bleeding/hospitalization outcomes in US patients with CKD. Anemia treatment was infrequent.

**Supplementary Information:**

The online version contains supplementary material available at 10.1186/s12882-022-02778-8.

## Introduction

Anemia of chronic kidney disease (CKD) is associated with increased risk of cardiovascular events and mortality, increased healthcare resource utilization (HCRU), and reduced health-related quality of life (HRQoL) [1–6]. While the prevalence of anemia increases with CKD severity [7–9], anemia also imparts a substantial healthcare burden in early-stage CKD [7].

Traditionally, management of anemia of CKD has relied on the variable use of iron replacement therapy and erythropoiesis-stimulating agents (ESAs), according to geographical region and CKD severity [10–12]. ESAs have demonstrated efficacy in correcting hemoglobin (Hb) levels, improving HRQoL, and reducing the need for red blood cell (RBC) transfusions [5, 13]. However, clinical trial data have showed inconsistent improvement in mortality, renal, or cardiovascular outcomes with ESA use in patients with CKD not on dialysis [14–16]. Notably, reports from several large randomized trials of an increased risk of mortality, thrombotic events, and cardiovascular outcomes in ESA-treated patients when targeted to normalize Hb [15–17] led the Food and Drug Administration and the Kidney Disease: Improving Global Outcomes (KDIGO) group to advise against using ESAs in patients with Hb levels > 10 g/dL [12, 18]. Recommendations from the European Renal Best Practice group suggest ESA initiation at Hb levels between 9 and 10 g/dL in high-risk patients (e.g. older patients and those with comorbidities), with the potential to initiate treatment at higher Hb levels in low-risk patients (e.g. younger patients with very few comorbidities) [19].

The optimal management of anemia of CKD remains controversial [3]. Marked reduction in ESA use has resulted in a shift towards less intensive therapy and lower Hb treatment targets in patients with anemia of CKD [8, 20, 21]. Notably, many patients with CKD not on dialysis do not receive any anemia treatment [7, 22], despite some evidence that untreated versus treated anemia is associated with higher HCRU and decreased HRQoL [5, 6]. Moreover, treating anemia of CKD can reduce the requirement for RBC transfusions, and thus associated risks from transfusion-related reactions and the development of alloantibodies [23].

Contemporary, real-world insights into the burden of anemia of CKD, HCRU, and long-term clinical outcomes for patients with CKD not on dialysis are needed to inform clinical practice. In particular, as novel treatment strategies for anemia with unique mechanisms of action and potentially improved cardiovascular safety emerge, information on treatment patterns and outcomes in patients with CKD will be particularly valuable to help physicians assess the benefits and risks of treatment on an individual patient basis. In this study, we used the Henry Ford Health System (HFHS) database to gather descriptive real-world information, including selected clinical outcomes of anemia, on US patients with CKD who were not receiving dialysis.

## Methods

### Study design

This was an observational cohort study using retrospective data from electronic healthcare records (EHRs) stored in the HFHS database. The study was approved by the HFHS Institutional Review Board, and all methods were carried out in accordance with relevant guidelines and regulations.

### Data sources

The HFHS is a large, integrated US healthcare delivery system that operates in five regions throughout Southeast Michigan and utilizes the Epic EHR system [24]. Inputs made at point-of-care are stored as structured data fields, and patients have a lifetime medical record number that is used across content areas including patient demographics, clinical encounters, medication orders, laboratory measures, and vital signs. Outpatient, emergency room, and inpatient encounters and procedures are captured using the International Classification of Diseases, Ninth or Tenth revision (ICD-9, ICD-10) codes, and Current Procedural Terminology, version 4 procedural codes. In addition to HFHS data, death certificates from the Michigan Department of Health and Human Services were used for mortality determination.

### Eligibility

Adults (aged ≥ 18 years) with CKD stages 3a–5, not on dialysis, and two estimated glomerular filtration rate (eGFR) measures < 60 mL/min/1.73m^2^ (determined using the CKD-epi equation [25]) ≥ 90 days apart between January 1, 2013 and December 31, 2017 (index date; time zero of follow-up from first eGFR measure) were eligible for inclusion.

Exclusion criteria included chronic renal replacement therapy occurring ≤ 6 months prior to index date, history of renal transplant, active cancer (identified by diagnosis and procedure codes for chemotherapy or radiation therapy), or bleeding episodes at baseline.

### Study outcomes

#### Anemia

Incidence rates per 100 person-years (PY) of anemia, defined as Hb < 10 g/dL (eligibility criterion for treatment with ESAs per the KDIGO 2012 guidelines [12]: the ‘gold standard’ for ESA treatment recommendations) in patients with known non-anemic status at baseline, overall, and according to baseline Hb level (10.0–10.9 g/dL, 11.0–12.0 g/dL, and > 12.0 g/dL). Baseline anemia was defined as Hb < 10 g/dL on at least one occasion within 6 months of the index date. Although this definition was for anemia more severe than the standard diagnosis threshold (Hb < 13.0 g/dL [men]; < 12.0 g/dL [women] [12]), in order to identify anemia eligible for active treatment, the term ‘anemia’ is used to describe this cohort for brevity.

#### Renal

Incidence rates per 100 PY of CKD progression: doubling of serum creatinine; 40% decrease in eGFR; kidney transplant; dialysis; and a composite of these individual outcomes.

#### Cardiovascular

Incidence rates per 100 PY of major adverse cardiovascular events (MACE), including MACE (first occurrence of the composite of all-cause mortality, non-fatal myocardial infarction, or non-fatal stroke), and MACE+ (first occurrence of MACE outcome, and including hospitalization for unstable angina or hospitalization for heart failure [hHF]).

#### Bleeding and hospitalization

Incidence rates per 100 PY of RBC transfusion, all-cause hospitalization, and hospitalization for active bleeding. Median duration of hospitalization was also calculated.

### Statistical analysis

The study baseline period was 6 months pre/post the index date. Patients were followed for up to 5 years, until December 31, 2018. Kaplan–Meier survival analysis determined the time to first event for study outcomes. Patients were censored at date of last contact with the HFHS (visit or eGFR laboratory result) or occurrence of a study outcome or death, whichever came first. Associations between baseline factors, including the presence of anemia, and outcomes at 1 and 5 years were explored using Cox proportional hazards models, simultaneously adjusted for baseline covariates.

As a sensitivity analysis, cumulative incidence function (CIF) of renal and cardiovascular outcomes was computed using competing risk analysis, with mortality as a competing event. Associations between baseline factors and 5-year outcomes were estimated using Fine and Gray’s proportional sub-distribution hazards regression models [26].

## Results

### Baseline characteristics

In total, 50,701 patients met the study eligibility criteria (Appendix Fig. S1) and 11,673 patients (23.0%) had anemia (Hb < 10 g/dL) at baseline (Table 1). Almost 60% of patients had stage 3a CKD and the proportions declined with increasing CKD severity (3.4% for stage 5 CKD). Anemia prevalence was greater with increasing CKD stage.

**Table 1:** Baseline characteristics

	All patients (*N* = 50,701)^a^	Patients with anemia (*N* = 11,673)	Patients without anemia (*N* = 35,033)
Sex, *n* (%)			
Female	28,450 (56.1)	7175 (61.5)	19,116 (54.6)
Mean age, years (SD)	72.0 (13.1)	71.2 (14.4)	72.2 (12.8)
Age group, *n* (%)			
< 50 years	2758 (5.4)	917 (7.9)	1715 (4.9)
50–59 years	5613 (11.1)	1425 (12.2)	3835 (10.9)
60–69 years	11,969 (23.6)	2645 (22.7)	8271 (23.6)
70–79 years	14,372 (28.3)	2913 (25.0)	10,043 (28.7)
≥ 80 years	15,989 (31.5)	3773 (32.3)	11,169 (31.9)
Race, *n* (%)			
African American	13,637 (26.9)	3559 (30.5)	8927 (25.5)
Asian	543 (1.1)	96 (0.8)	406 (1.2)
Hispanic	888 (1.8)	245 (2.1)	584 (1.7)
White	31,975 (63.1)	7085 (60.7)	22,530 (64.3)
Other/unknown	3658 (7.2)	688 (5.9)	2586 (7.4)
CKD stage, *n* (%)^b^			
3a	30,204 (59.6)	4979 (42.7)	22,444 (64.1)
3b	13,882 (27.4)	3548 (30.4)	9386 (26.8)
4	4880 (9.6)	2050 (17.6)	2597 (7.4)
5	1735 (3.4)	1096 (9.4)	606 (1.7)
Hb strata^c^ g/dL, *n* (%)			
< 8.0	4147 (8.2)	4147 (35.5)	–
8.0–9.9	7526 (14.8)	7526 (64.5)	–
10.0–12.0	15,196 (30.0)	–	15,196 (43.4)
> 12.0	19,837 (39.1)	–	19,837 (56.6)
Missing	3995 (7.9)	–	–
Anemia therapy, *n* (%)			
ESA	539 (1.1)	483 (4.1)	53 (0.2)
Iron (IV and oral)	4283 (8.4)	2825 (24.2)	1410 (4.0)
RBC transfusion	1306 (2.6)	1287 (11.0)	19 (0.1)
Any of the above	5219 (10.3)	3703 (31.7)	1468 (4.2)

^a^Included patients with anemia, without anemia, and those for whom no Hb measurement was available in the baseline period. The latter group of patients were excluded from the anemia and non-anemia cohorts

^b^CKD stages based on eGFR measurements (mL/min per 1.73 m^2^): 3a, 45–59; 3b, 30–44; 4, 15–29; 5, < 15

^c^Hb level/anemia status was determined from the lowest available Hb level during the 6 months pre/post index date

*CKD* chronic kidney disease, *ESA* erythropoiesis-stimulating agent, *Hb* hemoglobin, *IV* intravenous, *RBC* red blood cell, *SD* standard deviation

The proportion of patients with anemia receiving anemia therapies during the baseline period was 4.1, 24.2, 11.0, and 31.7% for ESAs, iron replacement therapy (intravenous and oral), RBC transfusion, and any therapy or combination of therapies, respectively. There was a low use of anemia therapies among patients without anemia at baseline (Hb ≥ 10 g/dL), which consisted mostly of iron replacement therapies (4.0%). Median duration of follow-up was 3.2 years (interquartile range: 1.9–4.7 years).

### Incident anemia

**Fig. 1:**
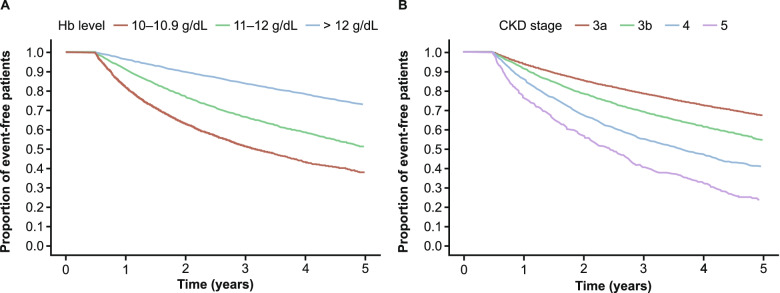
Kaplan–Meier curves for incident anemia according to baseline Hb level (**A**) and baseline CKD stage (**B**) in patients without anemia at baseline. The study baseline period comprised the 6 months before and after the index date, meaning that patients could not experience incident anemia during that period. *CKD* chronic kidney disease, *Hb* hemoglobin

Kaplan–Meier survival curves showing time to incident anemia in patients without anemia at baseline are shown in Fig. 1. Incidence rates of anemia were 7.4 and 9.7 per 100 PY at 1 year and 5 years of follow-up, respectively (Appendix Table S1). The risk of developing anemia was greatest in patients with baseline Hb 10.0–10.9 g/dL relative to Hb 11.0–12.0 g/dL and > 12 g/dL, and the risk increased with higher CKD stages (Fig. 1; Appendix Table S1).

### Renal outcomes

**Fig. 2:**
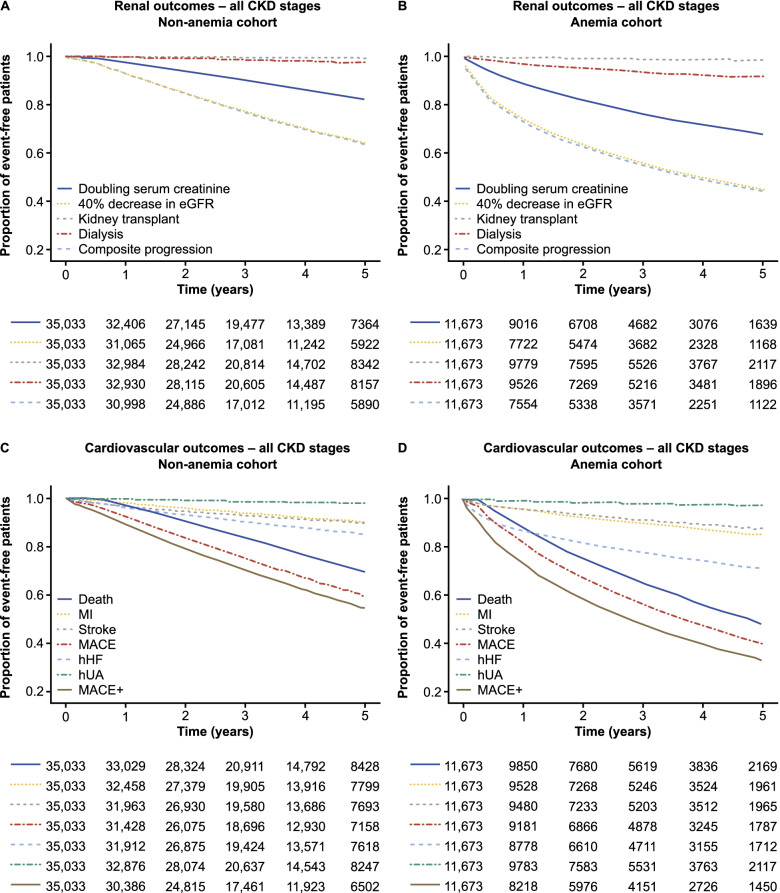
Kaplan–Meier curves for renal and cardiovascular outcomes in patients without anemia (**A**, **C**) and patients with anemia at baseline (**B**, **D**). Patients who died within 90 days of the index date were not included in outcomes analyses. Baseline anemia was defined as Hb < 10 g/dL, determined from the lowest available Hb level within 6 months of the index date. *CKD* chronic kidney disease, *eGFR* estimated glomerular filtration rate, *Hb* hemoglobin, *hHF* hospitalization for heart failure, *hUA* hospitalization for unstable angina, *MACE* major adverse cardiovascular events, *MACE+* first occurrence of MACE outcome, hUA or hHF, *MI* myocardial infarction

**Fig. 3:**
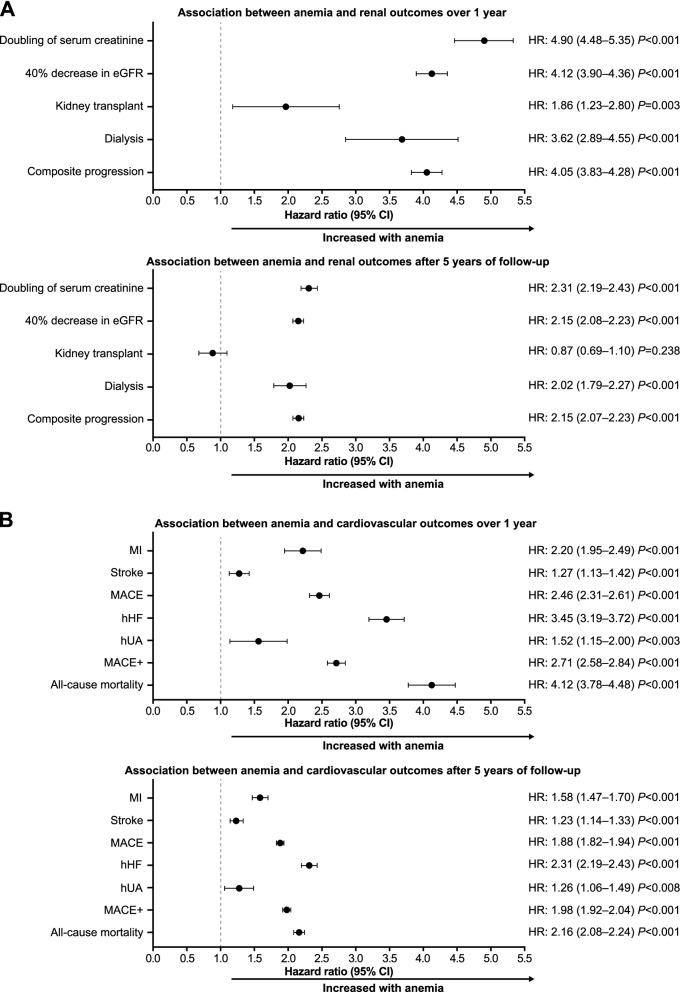
Multivariable HRs for renal (**A**) and cardiovascular (**B**) outcomes over 1 year and after 5 years of follow-up in patients with anemia versus those without anemia at baseline. Baseline anemia was defined as Hb < 10 g/dL, determined from the lowest available Hb level within 6 months of the index date. Multivariable HRs were calculated using Cox proportional hazards models, simultaneously adjusted for baseline covariates sex, age strata [< 50; 50–59; 60–69; 70–79; and 80+ years], ethnicity, CKD stage, and anemia. Patients whose anemia status was unknown were excluded from analyses using these models. *CI* confidence interval, *CKD* chronic kidney disease, *Hb* hemoglobin, *hHF* hospitalization for heart failure, *HR* hazard ratio, *hUA* hospitalization for unstable angina, *MACE* major adverse cardiovascular event, *MACE+* first occurrence of MACE outcome, hUA or hHF, *MI* myocardial infarction

Kaplan–Meier survival curves delineating renal outcomes are presented in Fig. 2A and B. Outcomes stratified by CKD stage are shown in Appendix Fig. S2. After 1 year, the incidence rates of the composite renal outcome were 33.8 and 7.5 per 100 PY in patients with and without anemia, respectively. At 5 years, the incidence rates were 21.3 and 8.8 per 100 PY in patients with and without anemia, respectively (Appendix Table S1). At both timepoints, the risk of renal outcomes was significantly higher in patients with anemia versus those without anemia at baseline (Fig. 3A).

### Cardiovascular outcomes

Kaplan–Meier survival curves delineating cardiovascular outcomes are shown in Fig. 2C and D. Outcomes stratified by CKD stage are shown in Appendix Fig. S3. After 1 year, the incidence rates of the composite outcomes of MACE and MACE+ were 19.5 and 31.6, respectively, per 100 PY in patients with anemia (Appendix Table S1). Of the component outcomes, incidence rates per 100 PY were highest for all-cause mortality and hHF (12.2 and 14.6, respectively). Incidence rates among patients without anemia were 8.0 and 11.5 per 100 PY for MACE and MACE+, respectively (Appendix Table S1).

After 5 years, incidence rates of MACE and MACE+ were 18.8 and 24.4 per 100 PY, respectively, in patients with anemia, and 9.9 and 12.0 per 100 PY, respectively, in patients without anemia (Appendix Table S1). Similar to after 1 year of follow-up, incidence rates per 100 PY were highest for all-cause mortality and hHF (14.1 and 8.4, respectively) in patients with anemia. The risk of all cardiovascular outcomes was significantly higher in patients with versus without anemia at baseline at 1 and 5 years (Fig. 3B).

### CIF curves for renal and cardiovascular outcomes

Findings from an analysis of renal and cardiovascular outcomes using CIF curves supported findings from the main analysis, showing an increased incidence of renal and cardiovascular outcomes in patients with versus without anemia at baseline (Appendix Fig. S4).

### Bleeding and hospitalization outcomes

Kaplan–Meier survival curves showing bleeding and hospitalization outcomes are presented in Appendix Fig. S5. The 1-year and 5-year incidence rates (per 100 PY) of outcomes were increased in patients with versus without anemia at baseline, and the risk of all outcomes was significantly higher in patients with versus without anemia at 1 and 5 years (Appendix Tables S1 and S2). The median lengths of all-cause hospitalization and hospitalization for active bleeding were increased in patients with versus without baseline anemia (Appendix Table S1).

### Association between baseline factors and clinical outcomes

Factors associated with increased risk of incident anemia at 1 and 5 years included female sex and increasing CKD stage (Appendix Fig. S6). Increasing CKD stage was also associated with increased risk of the composite renal outcome at both 1 and 5 years in the anemia and non-anemia cohorts, and with MACE and MACE+ after 5 years in both cohorts (Appendix Tables S3 and S4). Additional factors associated with increased risk of the composite renal and composite cardiovascular outcomes were male sex and white ethnicity (Appendix Tables S3 and S4). The relationship between age and clinical outcomes was less clear, with increasing age associated with reduced risk of the composite renal outcome, but an increased risk of MACE and MACE+ in both the anemia and non-anemia cohorts. Results from the competing risks analysis using Fine-Gray regression models were similar to the original analysis (Appendix Table S5).

In both the anemia and non-anemia cohorts, there were trends towards an association between male sex, white ethnicity, and increasing CKD stage with RBC transfusion and hospitalization outcomes (Appendix Table S2).

## Discussion

This retrospective cohort study investigated the baseline prevalence, use of anemia treatments, post-baseline incidence of anemia, and adverse clinical outcomes in patients with CKD not on dialysis at baseline, using data originating from primary and secondary care settings within a large US healthcare system. This analysis confirmed that potentially treatable anemia (i.e. Hb < 10 g/dL) was prevalent among patients with CKD but treated only infrequently (31.7% of the population with anemia at baseline were receiving any anemia treatment, predominantly iron replacement therapies). In addition, the cumulative incidence of anemia in patients with CKD without anemia at baseline was 7.4 and 38.8% at 1 year and 5 years, respectively, while the presence of anemia at baseline was independently associated with increased risks of adverse renal and cardiovascular outcomes at 1 and 5 years.

The baseline prevalence of severe anemia was 23.0% and increased with CKD stage, confirming previous reports [7, 9, 27, 28]. It is, however, difficult to compare with published prevalence estimates, given the differences in study populations, the time period in which prevalence is assessed, and in the thresholds used to define anemia. Among patients without anemia at baseline who developed anemia during follow-up, the decline in Hb levels occurred in parallel to a decline in eGFR over the same period. This finding emphasizes the importance of periodic screening of patients with CKD for anemia early in the course of the disease, as well as monitoring of Hb levels and symptoms of anemia as CKD progression occurs.

Despite the high prevalence of anemia in the study cohort, the frequency of prescriptions for anemia treatments, particularly ESAs, was relatively low. These findings concur with two other studies of patients with CKD stages 3–5 (either not on dialysis [1] or with dialysis status not specified [7]) using US datasets between 2007 and 2013. In both studies, < 40% of patients with anemia received treatment for this condition [1, 7]. Similarly, in a cross-sectional study of patients with CKD not on dialysis in China, approximately one-third of patients with anemia were receiving treatment with erythropoietin and/or iron products [27]. Other longitudinal studies have highlighted the potential undertreatment of anemia in patients with CKD and anemia. In one European study, there was a notable lack of anemia therapy modification, particularly with regard to iron supplementation, over 6 months in patients with Hb < 11 g/dL, despite unmet treatment goals [29]. Additionally, in a recent analysis from the prospective, multinational Chronic Kidney Disease Outcomes and Practice Patterns Study, the proportions of treatment-naive patients with stages 3–5 CKD and Hb < 10 g/dL prescribed any anemia therapy and ESAs during 12 months of follow-up were 40 and 28%, respectively [22].

The low ESA use observed in the present study may be attributable in part to the definition of anemia, which required at least one Hb value < 10 g/dL to be recorded in an outpatient setting, with exclusion of acute and common major causes of anemia, including active cancer and bleeding. Some patients may have subsequently had higher Hb values recorded, which is why they did not have an ESA prescription recorded at baseline. Other potential clinical factors include avoidance of associated cardiovascular events and uncertainty regarding the need for treatment prior to starting dialysis [1, 30–32]; the need for patients to meet certain thresholds in terms of Hb levels or other laboratory parameters before treatment is given; under-recognition of anemia in non-dialysis-dependent CKD as a treatable condition; operational challenges of administering parenteral drugs to patients not on dialysis; and cost. In particular, uncertainty regarding whether or not to treat patients with ESAs before dialysis initiation may be one reason for these findings. Studies have also highlighted substantial differences in anemia treatment practices, both between countries and also at the clinic level within individual countries [11, 28]. These differences may reflect several factors, including variation in adherence to different CKD management guidelines, selection of Hb target thresholds for ESA and iron prescribing, and use of anemia algorithms to guide clinical practice. Irrespective of the reason, the findings from this study suggest an incongruity between guidelines for the treatment of anemia [12, 19] and clinical practice, and a need for further exploration of optimal treatment practices for patients with anemia of CKD. This is particularly important in the context of evidence that suggests untreated anemia of CKD is associated with reduced HRQoL and increased HCRU [6]. However, it is important to acknowledge the potential risks associated with some currently available treatments for anemia, and the need for treating physicians to carefully balance the potential benefits of treatment against the risk of adverse events.

Incidence rates of adverse renal and cardiovascular outcomes in the study cohort were comparable with those seen in previous studies of patients with advanced CKD and at high cardiovascular risk [33, 34], and likely reflect the multimorbid patient population in this study. The incidence of these outcomes observed in patients with baseline anemia compared with those without anemia is consistent with findings from other studies that showed an increased risk of adverse outcomes, including mortality, renal events, and adverse cardiovascular outcomes in patients with CKD with versus without anemia [4, 35, 36]. In particular, our findings concur with those from a Danish cohort study of patients with severe CKD (eGFR < 30 mL/min/1.73m^2^), where the adjusted hazard ratios for incident dialysis, all-cause mortality, and MACE were markedly increased in non-dialysis-dependent patients with anemia (Hb < 12 [women] or < 13 g/dL [men]) versus without anemia, and increased with anemia severity [4]. As in the study by Toft et al. [4], the hazard ratios for renal and cardiovascular outcomes for patients with versus without anemia were higher at 1 year versus 5 years. This apparent attenuation in risk over time may in part reflect changes in both patient populations over time, where more ill patients died, and the survivors were selected for less severe disease. It could also be attributed to a greater likelihood of specialist care and an increased use of targeted renal and cardiovascular interventions in patients with the most severe disease. The risk of renal and cardiovascular outcomes was similar using both a Kaplan–Meier survival approach (to estimate the risk of events) and CIF (to estimate a patient’s risk of events prior to mortality); results using the latter method provide valuable additional insight from a population perspective, when taking into account mortality as a competing risk.

Whether anemia contributes directly or is merely associated with adverse renal and cardiovascular outcomes is unclear. On the one hand, it has been proposed that oxidative stress, inflammation, and diminished biological capacity contribute simultaneously to both anemia and adverse clinical outcomes without a clear linear relationship being present [37]. In particular, diminution of renal function leading to depressed erythropoietin production is a major cause of anemia [37]. Conversely, decreased oxygen delivery to tissues has been proposed to drive renal fibrosis through hypoxia-mediated signaling pathways [38], and chronic oxygen deprivation may also contribute to cardiac dysfunction by driving compensatory adaptations such as increased cardiac output [39].

Analysis of baseline factors associated with clinical outcomes showed that advancing CKD stage was generally associated with a greater risk of renal and cardiovascular events in all patients, regardless of whether they had anemia at baseline. Hazard ratios for outcomes assessed varied widely, which likely reflects differences in the risk of experiencing each outcome during the CKD disease course. Our findings also demonstrated an association between white ethnicity and an increased risk of renal, cardiovascular, and bleeding outcomes in both the anemia and non-anemia cohorts, although this was not evident at all time points, or for all outcomes. This contrasts with prior reports that suggested an increased rate of CKD progression associated with non-white ethnicity [40]. Notably, these studies differ in adjustment for baseline factors, including socioeconomic status, in analyses, necessitating caution in their interpretation.

Major strengths of this study include the large and diverse population, the extensive follow-up period, and comprehensive data source, including laboratory test results and prescription medication orders. Limitations include those that are inherent to many retrospective database analyses. Administrative data are not collected for research purposes and are subject to coding errors, and complete datasets were not available for all variables. Medications administered without a prescription (e.g. oral iron) were not captured; some patients were lost to follow-up; and there was no link between EHRs and retrospective claims. Information regarding the etiology of anemia was not available; thus, it was not possible to verify that all patients in the study had anemia attributable to CKD, although major causes of anemia other than CKD (e.g. bleeding and cancer) were excluded. Information on cause of death recorded on death certificates is not always informative, and this information was not readily accessible from other sources, due to Institutional Research Board regulations.

Although we adjusted for key baseline covariates when assessing associations between patient factors and clinical outcomes, there are other patient factors that may have also influenced the results, but were not well captured in the database, such as body mass index. Moreover, the anemia status of some patients (for example the small proportion of patients who were treated with ESAs at baseline) may have changed during the study follow-up period, which could have impacted clinical outcomes assessments.

## Conclusions

This study provides contemporary insight demonstrating that treatment of anemia of CKD was infrequent among patients with severe anemia meeting guideline criteria for treatment with ESAs. Anemia incidence was high among patients without baseline anemia (40% over 5 years). Moreover, patients with versus without anemia were at increased risk of adverse renal and cardiovascular outcomes.

These findings highlight the need to explore the underlying mechanisms of action and impact of newer treatments on adverse renal and cardiovascular outcomes in patients with anemia of CKD. They also indicate an opportunity for greater recognition of anemia of CKD as a significant complication, as it appears to be currently undertreated despite its association with an increased risk of adverse renal and cardiovascular outcomes, a situation that may improve via early screening and management.

Given that current treatment options for anemia of CKD are limited, the evaluation of newer, more effective, and well-tolerated therapies in real-world practice remains important.

## Supplementary Information

**Additional file 1: Appendix Table S1.** Clinical outcomes at 1 and 5 years. **Appendix Table S2.** Multivariable HRs for the association between baseline factors and bleeding outcomes over 1 year (A) and after 5 years (B) of follow-up in patients without anemia and in patients with anemia at baseline (bold). **Appendix Table S3.** Multivariable HRs for the association between baseline factors and renal outcomes over 1 year (A) and after 5 years (B) of follow-up in patients without anemia and in patients with anemia (bold). **Appendix Table S4.** Multivariable HRs for the association between baseline factors and cardiovascular outcomes over 1 year (A) and after 5 years (B) of follow-up in patients without anemia and in patients with anemia (bold). **Appendix Table S5.** Competing risks analysis of the association between baseline factors and renal (A) and cardiovascular (B) outcomes after 5 years of follow-up in patients without anemia and patients with anemia (bold). **Appendix Fig. S1.** Selection of the study cohort. **Appendix Fig. S2.** Kaplan–Meier curves for renal outcomes stratified by CKD stage over 5 years of follow-up, in patients without anemia (A) and patients with anemia at baseline (B). **Appendix Fig. S3.** Kaplan–Meier curves for cardiovascular outcomes stratified by CKD stage over 5 years of follow-up, in patients without anemia (A) and patients with anemia (B). **Appendix Fig. S4.** Competing risk models for 5-year outcomes (CIF). **Appendix Fig. S5.** Kaplan–Meier curves for bleeding and hospitalization outcomes in patients without anemia (A) and patients with anemia (B). **Appendix Fig. S6.** Multivariable HRs for the association between baseline factors and incident anemia over 1 year and after 5 years of follow-up.

## Data Availability

Data may be obtained from a third party and are not publicly available. De-identified participant data requests will be reviewed upon request by The Henry Ford Health System Regulatory Counsel. Please contact the corresponding author for more information.

## Abbreviations

CIF: Cumulative incidence function
CKD: Chronic kidney disease
eGFR: Estimated glomerular filtration rate
EHR: Electronic healthcare record
ESA: Erythropoiesis-stimulating agent
Hb: Hemoglobin
HCRU: Healthcare resource utilization
HFHS: Henry Ford Health System
hHF: Hospitalization for heart failure
HRQoL: Health-related quality of life
MACE: Major adverse cardiovascular events
MACE+: First occurrence of MACE outcome, and including hospitalization for unstable angina and hHF
MI: Myocardial infarction
PY: Person-years
RBC: Red blood cell
