# Supplementary material for: Epidemiology and outcomes in patients with anemia of CKD not on dialysis from a large US healthcare system database: a retrospective observational study
Source: BMC Nephrol. 2022 Apr 30;23:166. doi: 10.1186/s12882-022-02778-8 (PMC9055693; doi:10.1186/s12882-022-02778-8)
Supplement: Supplementary file 1 — Additional file 1: Appendix Table S1. Clinical outcomes at 1 and 5 years. Appendix Table S2. Multivariable HRs for the association between baseline factors and bleeding outcomes over 1 year (A) and after 5 years (B) of follow-up in patients without anemia and in patients with anemia at baseline (bold). Appendix Table S3. Multivariable HRs for the association between baseline factors and renal outcomes over 1 year (A) and after 5 years (B) of follow-up in patients without anemia and in patients with anemia (bold). Appendix Table S4. Multivariable HRs for the association between baseline factors and cardiovascular outcomes over 1 year (A) and after 5 years (B) of follow-up in patients without anemia and in patients with anemia (bold). Appendix Table S5. Competing risks analysis of the association between baseline factors and renal (A) and cardiovascular (B) outcomes after 5 years of follow-up in patients without anemia and patients with anemia (bold). Appendix Fig. S1. Selection of the study cohort. Appendix Fig. S2. Kaplan–Meier curves for renal outcomes stratified by CKD stage over 5 years of follow-up, in patients without anemia (A) and patients with anemia at baseline (B). Appendix Fig. S3. Kaplan–Meier curves for cardiovascular outcomes stratified by CKD stage over 5 years of follow-up, in patients without anemia (A) and patients with anemia (B). Appendix Fig. S4. Competing risk models for 5-year outcomes (CIF). Appendix Fig. S5. Kaplan–Meier curves for bleeding and hospitalization outcomes in patients without anemia (A) and patients with anemia (B). Appendix Fig. S6. Multivariable HRs for the association between baseline factors and incident anemia over 1 year and after 5 years of follow-up. [file 12882_2022_2778_MOESM1_ESM.docx]

# Supplementary material

Appendix Table S1. Clinical outcomes at 1 and 5 years

|  | **Patients without anemia** | | **Patients with anemia^a^** | |
| --- | --- | --- | --- | --- |
|  | **1 year** | **5 years** | **1 year** | **5 years** |
| **Incident anemia outcomes, *n* (incidence rate per 100 PY)** | | | | |
| All patients without baseline anemia (*n* = 35,033) | 2515 (7.4) | 10,106 (9.7) | – | – |
| Hb 10–10.9 g/dL | 1083 (18.3) | 3129 (20.4) | – |  |
| Hb 11–12 g/dL | 737 (8.7) | 3205 (12.9) | – | – |
| Hb > 12 g/dL | 695 (3.6) | 3772 (5.9) | – | – |
| **Renal outcomes, *n* (incidence rate per 100 PY)** | | | | |
| Doubling of serum creatinine | 863 (2.5) | 4146 (3.6) | 1274 (12.3) | 2696 (9.0) |
| 40% decrease in eGFR | 2427 (7.3) | 9117 (8.7) | 2947 (31.4) | 5084 (20.2) |
| Kidney transplant | 45 (0.1) | 166 (0.1) | 72 (0.7) | 140 (0.4) |
| Dialysis | 105 (0.3) | 576 (0.5) | 352 (3.3) | 709 (2.2) |
| **Composite outcome^b^** | **2496 (7.5)** | **9207 (8.8)** | **3125 (33.8)** | **5240 (21.3)** |
| **Cardiovascular outcomes, *n* (incidence rate per 100 PY)** | | | | |
| All-cause mortality | 1009 (2.9) | 7798 (6.5) | 1331 (12.2) | 4812 (14.1) |
| MI | 678 (2.0) | 2446 (2.1) | 463 (4.3) | 1119 (3.5) |
| Stroke | 1175 (3.5) | 2713 (2.4) | 460 (4.3) | 959 (3.0) |
| **MACE^c^** | **2667 (8.0)** | **10,947 (9.9)** | **2040 (19.5)** | **5783 (18.8)** |
| hHF | 1359 (4.0) | 3482 (3.4) | 1474 (14.6) | 2505 (8.4) |
| hUA | 165 (0.5) | 530 (0.5) | 81 (0.7) | 196 (0.6) |
| **MACE+^d^** | **3762 (11.5)** | **12,617 (12.0)** | **3058 (31.6)** | **6656 (24.4)** |
| **Bleeding outcomes, *n* (incidence rate per 100 PY)** | | | | |
| RBC transfusions | 91 (0.3) | 467 (0.5) | 2601 (13.2) | 7449 (12.5) |
| All-cause hospitalizations | 4043 (15.6) | 9293 (11.5) | 7918 (47.4) | 17,068 (41.6) |
| Hospitalizations for active bleeding | 11 (0.04) | 78 (0.1) | 135 (0.7) | 563 (0.8) |
| **Median (IQR) length of hospital stay, days** | | | | |
| All-cause hospitalizations | 4 (2–8) | | 11 (5–23) | |
| Hospitalizations for active bleeding | 5 (2–7) | | 7 (4–13) | |

^a^Defined as patients with baseline anemia (< 10 g/dL) within 6 months of the index date for all outcomes with the exception of bleeding outcomes where the anemia cohort included both incident and prevalent cases.
^b^Composite of doubling of serum creatinine, 40% decrease in eGFR, kidney transplant and dialysis.
^c^Composite of all-cause mortality, non-fatal MI, or non-fatal stroke.
^d^Composite of MACE outcome, hUA, or hHF.

*CKD* chronic kidney disease*, eGFR* estimated glomerular filtration rate, *HF* heart failure, *hHF* hospitalization for heart failure, *hUA* hospitalization for unstable angina, *IQR* interquartile range, *MACE* major adverse cardiovascular events, *MACE*+ first occurrence of MACE outcome, hUA or hHF, *MI* myocardial infarction, *PY* person-years; *RBC* red blood cell.

Appendix Table S2. Multivariable HRs for the association between baseline factors and bleeding outcomes over 1 year (A) and after 5 years (B) of follow-up in patients without anemia and in patients with anemia^a^ at baseline (bold)

**A**

| **Variable** | **RBC transfusion** | **All-cause hospitalization** | **Hospitalization for active bleeding** |
| --- | --- | --- | --- |
| Age | 1.14 (0.95–1.39); *p* = 0.167 **0.93 (0.90–0.96); *p* < 0.001** | 1.02 (0.99–1.05); *p* = 0.108 **0.99 (0.98–1.01); *p* = 0.528** | 1.04 (0.64–1.82); *p* = 0.878 **0.91 (0.79–1.05); *p* = 0.182** |
| Male (vs. female) | 1.82 (1.20–2.81); *p* = 0.005 **1.07 (0.99–1.16); *p* = 0.096** | 1.21 (1.14–1.29); *p* < 0.001 **1.02 (0.98–1.07); *p* = 0.330** | 1.06 (0.32–3.39); *p* = 0.919 **1.27 (0.91–1.79); *p* = 0.163** |
| Non-white (vs. white) ethnicity | 0.92 (0.58–1.42); *p* = 0.714 **0.88 (0.81–0.96); *p* = 0.002** | 0.84 (0.78–0.89); *p* < 0.001  **0.86 (0.82–0.90); *p* < 0.001** | 1.08 (0.30–3.46); *p* = 0.898 **0.87 (0.61–1.24); *p* = 0.441** |
| Stage 5 (vs. 3a) | 0.64 (0.005–4.46); *p* = 0.736  **1.16 (0.99–1.35); *p* = 0.063** | 1.57 (1.19–2.06); *p* = 0.001 **1.26 (1.16–1.38); *p* < 0.001** | 7.03 (0.05–64.81); *p* = 0.305  **0.91 (0.43–1.93); *p* = 0.812** |
| Stage 4 (vs. 3a) | 1.12 (0.41–2.46); *p* = 0.798 **1.11 (0.99–1.24); *p* = 0.065** | 1.37 (1.21–1.55); *p* < 0.001 **1.15 (1.08–1.22); *p* < 0.001** | 7.96 (1.84–29.98); *p* = 0.008 **1.31 (0.82–2.10); *p* = 0.264** |
| Stage 3b (vs. 3a) | 1.19 (0.73–1.88); *p* = 0.476  **1.00 (0.91–1.10); *p* = 0.995** | 1.24 (1.16–1.33); *p* < 0.001 **1.06 (1.01–1.12); *p* = 0.019** | 1.78 (0.40–6.80); *p* = 0.420 **1.10 (0.74–1.63); *p* = 0.651** |
| Anemia | 40.30 (32.67–49.71); *p* < 0.001 **–** | 2.87 (2.76–2.99); *p* < 0.001 **–** | 16.31 (8.77–30.31); *p* < 0.001 **–** |

**B**

| **Variable** | **RBC transfusion** | **All-cause hospitalization** | **Hospitalization for active bleeding** |
| --- | --- | --- | --- |
| Age | 1.19 (1.09–1.30); *p* < 0.001 **1.00 (0.98–1.02); *p* = 0.947** | 1.04 (1.02–1.06); *p* < 0.001 **1.00 (0.99–1.02); *p* = 0.716** | 1.02 (0.84–1.25); *p* = 0.826 **0.96 (0.90–1.03); *p* = 0.245** |
| Male (vs. female) | 1.45 (1.20–1.74); *p* < 0.001 **1.13 (1.07–1.18); *p* < 0.001** | 1.20 (1.16–1.25); *p* < 0.001 **1.08 (1.05–1.12); *p* < 0.001** | 1.53 (0.98–2.41); *p* = 0.064 **1.43 (1.21–1.69); *p* < 0.001** |
| Non-white (vs. white) ethnicity | 1.01 (0.83–1.23); *p* < 0.904 **0.87 (0.83–0.91); *p* < 0.001** | 0.86 (0.82–0.89); *p* < 0.001  **0.87 (0.84–0.90); *p* < 0.001** | 0.70 (0.43–1.16); *p* = 0.167 **0.93 (0.78–1.10); *p* = 0.389** |
| Stage 5 (vs. 3a) | 2.02 (0.90–4.55); *p* = 0.089  **1.15 (1.04–1.26); *p* = 0.005** | 1.35 (1.10–1.65); *p =* 0.004 **1.09 (1.02–1.16); *p* = 0.007** | 1.85 (0.25–13.51); *p* = 0.544  **0.73 (0.49–1.09); *p* = 0.123** |
| Stage 4 (vs 3a) | 2.02 (1.47–2.79); *p* < 0.001 **1.17 (1.09–1.25); *p* < 0.001** | 1.29 (1.19–1.41); *p* < 0.001 **1.05 (1.00–1.10); *p* = 0.032** | 2.34 (1.11–4.96); *p* = 0.026 **1.16 (0.91–1.47); *p* = 0.229** |
| Stage 3b (vs. 3a) | 1.24 (1.00–1.53); *p* = 0.047  **1.03 (0.97–1.08); *p* = 0.305** | 1.13 (1.08–1.19); *p* < 0.001 **0.99 (0.96–1.03); *p* = 0.594** | 1.31 (0.78–2.19); *p* = 0.315 **1.01 (0.83–1.22); *p* = 0.958** |
| Anemia | 25.75 (23.44–28.29); *P* < 0.001 **–** | 3.49 (3.40–3.59); *p* < 0.001 **–** | 10.50 (8.26–13.34); *p* < 0.001 **–** |

Multivariable HRs were calculated using Cox proportional hazards models, simultaneously adjusted for baseline covariates sex, age strata [<50; 50–59; 60–69; 70–79; and 80+ years], ethnicity, CKD stage, and anemia. Patients whose anemia status was unknown were excluded from analyses using these models.
^a^Defined as Hb < 10 g/dL and including both incident and prevalent cases.

*CI* confidence interval, *CKD* chronic kidney disease, *Hb* hemoglobin, *HR* hazard ratio, *RBC* red blood cell.

Appendix Table S3. Multivariable HRs for the association between baseline factors and renal outcomes over 1 year (A) and after 5 years (B) of follow-up in patients without anemia and in patients with anemia^a^ (bold)

**A**

| **Variable** | **Doubling of creatinine** | **40% decrease  in eGFR** | **Kidney transplant** | **Dialysis** | **Composite outcome^c^** |
| --- | --- | --- | --- | --- | --- |
| Age | 0.92 (0.87–0.98) **0.85 (0.81–0.88)** | 0.97 (0.94–1.01) **0.89 (0.87–0.92)** | 0.48 (0.37–0.62)  **0.40 (0.32–0.50)** | 0.72 (0.61–0.84) **0.76 (0.70–0.82)** | 0.96 (0.93–1.00) **0.87 (0.85–0.90)** |
| Male (vs. female) | 1.17 (1.03–1.34) **1.14 (1.02–1.28)** | 1.10 (1.02–1.19) **1.16 (1.08–1.25)** | 1.88 (0.99–3.77) **1.63 (1.02–2.61)** | 1.59 (1.06–2.40) **1.45 (1.18–1.79)** | 1.12 (1.03–1.21) **1.19 (1.11–1.30)** |
| Non-white (vs. white) ethnicity | 0.75 (0.65–0.87) **1.01 (0.89–1.14)** | 0.78 (0.72–0.86) **0.98 (0.90–1.06)** | 2.20 (1.15–4.51)  **3.74 (2.13–6.56)** | 0.53 (0.36–0.78) **0.52 (0.42–0.64)** | 0.80 (0.73–0.87) **0.98 (0.91–1.10)** |
| Stage 5 (vs. 3a) | 1.15 (0.72–1.85) **0.48 (0.37–0.61)** | 2.13 (1.70–2.66) **0.94 (0.83–1.07)** | ^b^ **91.04 (22.12–374.71)** | 149.22 (77.40–287.66) **47.53 (29.66–76.16)** | 3.90 (3.28–4.63) **1.62 (1.45–1.80)** |
| Stage 4 (vs. 3a) | 1.47 (1.17–1.84) **1.03 (0.88–1.19)** | 1.50 (1.31–1.72) **0.99 (0.90–1.10)** | ^b^  **13.08 (2.86–59.70)** | 17.64 (8.59–36.22) **11.11 (6.75–18.28)** | 1.54 (1.35–1.77) **1.05 (0.95–1.20)** |
| Stage 3b (vs. 3a) | 1.16 (0.99–1.35) **1.00 (0.88–1.14)** | 1.16 (1.05–1.27) **0.98 (0.90–1.07)** | **^b^**  **3.55 (0.65–19.39)** | 2.35 (0.97–5.68) **2.13 (1.17–3.87)** | 1.16 (1.06–1.28) **0.99 (0.91–1.10)** |
| Index anemia | 4.90 (4.48–5.35) **–** | 4.12 (3.90–4.36) **–** | 1.86 (1.23–2.80) **–** | 3.62 (2.89–4.55) **–** | 4.05 (3.83–4.28) **–** |

**B**

| **Variable** | **Doubling of creatinine** | **40% decrease  in eGFR** | **Kidney transplant** | **Dialysis** | **Composite outcome** |
| --- | --- | --- | --- | --- | --- |
| Age | 0.90 (0.88–0.93) **0.87 (0.85–0.90)** | 1.00 (0.98–1.01) **0.94 (0.92–0.96)** | 0.44 (0.38–0.51) **0.45 (0.39–0.53)** | 0.67 (0.63–0.71) **0.72 (0.68–0.77)** | 0.99 (0.97–1.01) **0.92 (0.90–0.94)** |
| Male (vs. female) | 1.21 (1.14–1.29) **1.11 (1.02–1.20)** | 1.13 (1.08–1.18) **1.13 (1.07–1.20)** | 1.77 (1.27–2.48) **1.26 (0.91–1.76)** | 1.70 (1.43–2.02) **1.34 (1.15–1.55)** | 1.13 (1.09–1.18) **1.16 (1.10–1.23)** |
| Non-white (vs. white) ethnicity | 0.70 (0.66–0.75) **0.83 (0.76–0.90)** | 0.80 (0.76–0.84) **0.87 (0.82–0.92)** | 1.94 (1.38–2.72) **2.20 (1.54–3.15)** | 0.50 (0.42–0.59) **0.54 (0.46–0.62)** | 0.81 (0.77–0.84) **0.88 (0.83–0.93)** |
| Stage 5 (vs. 3a) | 1.38 (1.12–1.70) **0.64 (0.55–0.75)** | 2.05 (1.80–2.33) **0.96 (0.87–1.06)** | 538.70 (196.63–1475.88) **68.71 (27.83–169.60)** | 34.31 (26.60–44.25) **15.60 (12.28–19.81)** | 3.22 (2.88–3.61) **1.51 (1.38–1.65)** |
| Stage 4 (vs. 3a) | 1.99 (1.80–2.19) **1.29 (1.16–1.42)** | 1.69 (1.58–1.81) **1.07 (0.99–1.16)** | 113.98 (41.18–315.48) **17.40 (6.78–44.68)** | 16.17 (12.87–20.31) **7.02 (5.49–8.96)** | 1.72 (1.61–1.85) **1.11 (1.02–1.20)** |
| Stage 3b (vs. 3a) | 1.32 (1.23–1.41) **1.05 (0.96–1.15)** | 1.18 (1.13–1.24) **1.00 (0.94–1.07)** | 14.64 (4.95–43.26) **2.83 (0.93–8.65)** | 3.11 (2.41–4.01) **1.66 (1.24–2.22)** | 1.18 (1.13–1.24) **1.01 (0.95–1.08)** |
| Index anemia | 2.31 (2.19–2.43) **–** | 2.15 (2.08–2.23) **–** | 0.87 (0.69–1.10) **–** | 2.02 (1.79–2.27) **–** | 2.15 (2.07–2.23) **–** |

Multivariable HRs were calculated using Cox proportional hazards models, simultaneously adjusted for baseline covariates sex, age strata [<50; 50–59; 60–69; 70–79; and 80+ years], ethnicity, CKD stage, and anemia. Patients whose anemia status was unknown were excluded from analyses using these models.
^a^Defined as Hb < 10 g/dL.
^b^No transplant events occurred in patients with stage 3a CKD in the non-anemia cohort at 1-year follow-up, so CKD stage 5 was used as the reference group. HR (95% CI) for transplant were as follows. Stage 3a (vs 5): 0 (0–0.01), *p* < 0.001; stage 3b (vs 5): 0.02 (0.007–0.05), *p* < 0.001; stage 4 (vs 5): 0.13 (0.06–0.26), *p* < 0.001.
^c^Composite of doubling of serum creatinine, 40% decrease in eGFR, kidney transplant and dialysis.

*CI* confidence interval, *CKD* chronic kidney disease, *eGFR* estimated glomerular filtration rate, *Hb* hemoglobin, *HR* hazard ratio.

Appendix Table S4. Multivariable HRs for the association between baseline factors and cardiovascular outcomes over 1 year (A) and after 5 years (B) of follow-up in patients without anemia and in patients with anemia^a^ (bold)

**A**

| **Variable** | **All-cause mortality** | **MI** | **Stroke** | **MACE^b^** | **hHF** | **hUA** | **MACE+^,c^** |
| --- | --- | --- | --- | --- | --- | --- | --- |
| Age | 1.79 (1.67–1.92) **1.31 (1.24–1.37)** | 0.83 (0.73–0.94) **1.24 (1.14–1.34)** | 1.18 (1.12–1.24) **1.14 (1.06–1.23)** | 1.32 (1.27–1.37) **1.23 (1.19–1.28)** | 1.08 (1.03–1.13) **1.09 (1.05–1.14)** | 0.88 (0.82–0.94) **0.94 (0.79–1.11)** | 1.20 (1.17–1.24) **1.16 (1.12–1.19)** |
| Male (vs. female) | 1.43 (1.26–1.63) **1.28 (1.15–1.43)** | 1.81 (1.32–2.48) **1.21 (1.01–1.46)** | 1.03 (0.92–1.16) **1.10 (0.91–1.33)** | 1.27 (1.18–1.37) **1.21 (1.10–1.32)** | 1.40 (1.25–1.55) **1.15 (1.04–1.28)** | 1.43 (1.20–1.70) **1.13 (0.73– 1.77)** | 1.32 (1.24–1.41) **1.18 (1.10–1.27)** |
| Non-white (vs. white) ethnicity | 1.21 (1.03–1.42) **1.38 (1.21–1.57)** | 0.57 (0.41–0.78) **1.08 (0.88–1.34)** | 0.56 (0.50–0.64) **0.49 (0.41–0.59)** | 0.78 (0.71–0.85) **1.01 (0.91–1.11)** | 0.47 (0.42–0.52) **0.62 (0.56–0.69)** | 0.72 (0.60–0.86) **0.54 (0.35–0.85)** | 0.64 (0.60–0.69) **0.79 (0.74–0.86)** |
| Stage 5 (vs. 3a) | 2.72 (1.82–4.07) **1.41 (1.16–1.72)** | 0.52 (0.13–2.11) **0.96 (0.67–1.38)** | 0.57 (0.31–1.03) **0.58 (0.39–0.85)** | 1.27 (0.95–1.71) **1.06 (0.89–1.25)** | 0.64 (0.39–1.07) **0.80 (0.65–0.98)** | 0.72 (0.36–1.45) **0.85 (0.39–1.84)** | 0.98 (0.75–1.27) **0.94 (0.82–1.07)** |
| Stage 4 (vs. 3a) | 2.14 (1.77–2.60) **1.27 (1.09–1.47)** | 1.52 (0.94–2.48) **1.20 (0.93–1.53)** | 0.81 (0.64–1.04) **0.74 (0.57–0.97)** | 1.28 (1.11–1.47) **1.12 (0.99–1.26)** | 1.51 (1.26–1.81) **1.20 (1.05–1.38)** | 1.23 (0.91–1.66) **0.77 (0.40–1.47)** | 1.33 (1.19–1.49) **1.13 (1.02–1.24)** |
| Stage 3b (vs. 3a) | 1.48 (1.29–1.70) **1.21 (1.06–1.38)** | 0.96 (0.66–1.39) **1.06 (0.85–1.32)** | 1.12 (0.98–1.27) **0.89 (0.72–1.10)** | 1.24 (1.14–1.35) **1.12 (1.01–1.24)** | 1.32 (1.17–1.49) **1.11 (0.98–1.25)** | 0.93 (0.75–1.14) **0.91 (0.54–1.54)** | 1.25 (1.16–1.34) **1.08 (1.00–1.18)** |
| Index anemia | 4.12 (3.78–4.48) **–** | 2.20 (1.95–2.49) **–** | 1.27 (1.13–1.42) **–** | 2.46 (2.31–2.61) **–** | 3.45 (3.19–3.72) **–** | 1.52 (1.15–2.00) **–** | 2.71 (2.58–2.84) **–** |

**B**

| **Variable** | **All-cause mortality** | **MI** | **Stroke** | **MACE^b^** | **HF** | **hUA** | **MACE+^,c^** |
| --- | --- | --- | --- | --- | --- | --- | --- |
| Age | 1.64 (1.60–1.68) **1.39 (1.36–1.43)** | 1.21 (1.16–1.25) **1.22 (1.16–1.29)** | 1.21 (1.17–1.25) **1.16 (1.10–1.23)** | 1.42 (1.39–1.44) **1.31 (1.28–1.35)** | 1.17 (1.14–1.21) **1.15 (1.11–1.19)** | 0.88 (0.82–0.94) **0.95 (0.85–1.06)** | 1.33 (1.30–1.35) **1.25 (1.22–1.27)** |
| Male (vs. female) | 1.31 (1.25–1.37) **1.26 (1.19–1.34)** | 1.31 (1.20–1.41) **1.24 (1.10–1.40)** | 1.04 (0.96–1.12) **1.02 (0.89–1.16)** | 1.25 (1.21–1.30) **1.22 (1.15–1.28)** | 1.31 (1.23–1.40) **1.14 (1.05–1.24)** | 1.43 (1.20–1.70) **1.09 (0.82–1.46)** | 1.26 (1.22–1.31) **1.21 (1.15–1.27)** |
| Non-white (vs. white) ethnicity | 1.05 (0.99–1.11) **1.18 (1.10–1.26)** | 1.10 (1.00–1.21) **1.19 (1.04–1.36)** | 0.65 (0.60–0.70) **0.59 (0.52–0.67)** | 0.93 (0.89–0.97) **1.03 (0.98–1.10)** | 0.68 (0.64–0.73) **0.72 (0.66–0.78)** | 0.72 (0.60–0.86) **0.70 (0.53–0.94)** | 0.86 (0.82–0.89) **0.90 (0.85–0.95)** |
| Stage 5 (vs. 3a) | 2.64 (2.27–3.06) **1.53 (1.38–1.69)** | 1.80 (1.38–2.36) **1.44 (1.17–1.77)** | 0.65 (0.45–0.95) **0.75 (0.58–0.97)** | 1.79 (1.57–2.05) **1.34 (1.22–1.48)** | 1.27 (0.99–1.62) **1.00 (0.86–1.17)** | 0.72 (0.36–1.45) **1.27 (0.83–1.95)** | 1.70 (1.50–1.92) **1.25 (1.14–1.36)** |
| Stage 4 (vs. 3a) | 2.00 (1.85–2.15) **1.36 (1.26–1.47)** | 1.58 (1.38–1.81) **1.45 (1.23–1.69)** | 0.98 (0.84–1.14) **0.87 (0.73–1.05)** | 1.63 (1.53–1.74) **1.28 (1.19–1.37)** | 1.70 (1.53–1.89) **1.31 (1.18–1.46)** | 1.23 (0.91–1.66) **0.69 (0.45–1.07)** | 1.61 (1.51–1.71) **1.26 (1.18–1.35)** |
| Stage 3b (vs. 3a) | 1.48 (1.41–1.55) **1.23 (1.15–1.32)** | 1.24 (1.13–1.36) **1.10 (0.95–1.27)** | 1.12 (1.03–1.22) **0.95 (0.82–1.10)** | 1.34 (1.28–1.40) **1.19 (1.12–1.26)** | 1.38 (1.28–1.48) **1.20 (1.09–1.31)** | 0.93 (0.75–1.14) **0.71 (0.50–1.02)** | 1.32 (1.27–1.37) **1.16 (1.10–1.23)** |
| Index anemia | 2.16 (2.08–2.24) **–** | 1.58 (1.47–1.70) **–** | 1.23 (1.14–1.33) **–** | 1.88 (1.82–1.94) **–** | 2.31 (2.19–2.43) **–** | 1.26 (1.06–1.49) **–** | 1.98 (1.92–2.04) **–** |

Multivariable HRs were calculated using Cox proportional hazards models, simultaneously adjusted for baseline covariates sex, age strata [<50; 50–59; 60–69; 70–79; and 80+ years], ethnicity, CKD stage, and anemia. Patients whose anemia status was unknown were excluded from analyses using these models.
^a^Defined as Hb < 10 g/dL.

^b^Composite of all-cause mortality, non-fatal MI, or non-fatal stroke.

^c^Composite of MACE outcome, hUA, or hHF.

*CI* confidence interval, *CKD* chronic kidney disease, *Hb* hemoglobin, *hHF* hospitalization for heart failure, *HR* hazard ratio, *hUA* hospitalization for unstable angina, *MACE* major adverse cardiovascular events, *MI* myocardial infarction.

Appendix Table S5. Competing risks analysis of the association between baseline factors and renal (A) and cardiovascular (B) outcomes after 5 years of follow-up in patients without anemia and patients with anemia^a^ (bold)

**A**

| **Variable** | **Composite outcome^b^** |
| --- | --- |
| Age | 0.95 (0.94–0.97), *p* < 0.001  **0.89 (0.87–0.91), *p* < 0.001** |
| Male (vs. female) | 1.11 (1.06–1.15), *p* < 0.001  **1.12 (1.06–1.19), *p* < 0.001** |
| Non-white (vs. white) ethnicity | 0.80 (0.76–0.84), *p* < 0.001  **0.86 (0.81–0.91), *p* < 0.001** |
| Stage 5 (vs. 3a) | 2.86 (2.54–3.22), *p* < 0.001  **1.35 (1.23–1.48), *p <* 0.001** |
| Stage 4 (vs. 3a) | 1.58 (1.47–1.69), *p* < 0.001  **1.04 (0.97–1.13), *p* = 0.28** |
| Stage 3b (vs. 3a) | 1.14 (1.09–1.20), *p* < 0.001  **0.98 (0.92–1.05), *p* = 0.55** |

**B**

| **Variable** | **MI** | **Stroke** | **hHF** | **hUA** |
| --- | --- | --- | --- | --- |
| Age | 1.15 (1.11–1.19), *p* < 0.001  **1.15 (1.09–1.20), *p* < 0.001** | 1.17 (1.13–1.21), *p* < 0.001  **1.11 (1.05–1.16), *p* < 0.001** | 1.13 (1.10–1.16), *p* < 0.001  **1.10 (1.07–1.14), *p* < 0.001** | 0.84 (0.79–0.90), *p* < 0.001  **0.90 (0.82–0.99), *p* = 0.036** |
| Male (vs. female) | 1.27 (1.17–1.37), *p* < 0.001  **1.17 (1.04–1.32), *p* = 0.008** | 1.01 (0.94–1.09), *p* = 0.740  **0.97 (0.85–1.11). *p* = 0.67** | 1.28 (1.20–1.37), *p* < 0.001  **1.10 (1.01–1.19), *p* = 0.024** | 1.39 (1.17–1.65), *p* < 0.001  **1.04 (0.78–1.39), *p* = 0.77** |
| Non-white (vs. white) ethnicity | 1.09 (0.99–1.19), *p* = 0.089  **1.14 (0.99–1.30), *p* = 0.06** | 0.64 (0.59–0.70), *p* < 0.001  **0.57 (0.50–0.65), *p* < 0.001** | 0.67 (0.63–0.72), *p* > 0.001 **0.69 (0.64–0.75), *p* < 0.001** | 0.71 (0.59–0.85), *p* < 0.001  **0.68 (0.50–0.91), *p* = 0.010** |
| Stage 5 (vs. 3a) | 1.63 (1.25–2.14), *p* < 0.001  **1.31 (1.07–1.62), *p* = 0.01** | 0.61 (0.41–0.89), *p* = 0.010  **0.70 (0.54–0.90), *p* = 0.006** | 1.15 (0.90–1.47), *p* = 0.250  **0.93 (0.80–1.09), *p* = 0.37** | 0.67 (0.33–1.35), *p* = 0.26  **1.19 (0.77–1.83), *p* = 0.43** |
| Stage 4 (vs. 3a) | 1.44 (1.26–1.65), *p* < 0.001  **1.35 (1.15–1.58), *p* < 0.001** | 0.91 (0.78–1.06), *p* = 0.230  **0.83 (0.69–0.99), *p* = 0.043** | 1.58 (1.42–1.76), *p* < 0.001  **1.26 (1.13–1.40), *p* < 0.001** | 1.14 (0.84–1.55), *p* = 0.39  **0.66 (0.43–1.02), *p* = 0.058** |
| Stage 3b (vs. 3a) | 1.19 (1.09–1.30), *p* < 0.001  **1.05 (0.91–1.21), *p* = 0.53** | 1.08 (1.00–1.18), *p* = 0.066  **0.92 (0.79–1.07), *p* = 0.27** | 1.34 (1.24–1.43), *p* < 0.001  **1.17 (1.06–1.28), *p* = 0.001** | 0.90 (0.73–1.10), *p* = 0.30  **0.69 (0.48–0.99), *p* = 0.041** |

HRs and 95% CIs were estimated using Fine-Gray competing risk models.

^a^Defined as Hb <10 g/dL.

^b^Composite of doubling of serum creatinine, 40% decrease in eGFR, kidney transplant and dialysis.

*CI* confidence interval, *hHF* hospitalization for heart failure, *HR* hazard ratio, *hUA* hospitalization for unstable angina, *MI* myocardial infarction.

Appendix Fig. S1 Selection of the study cohort


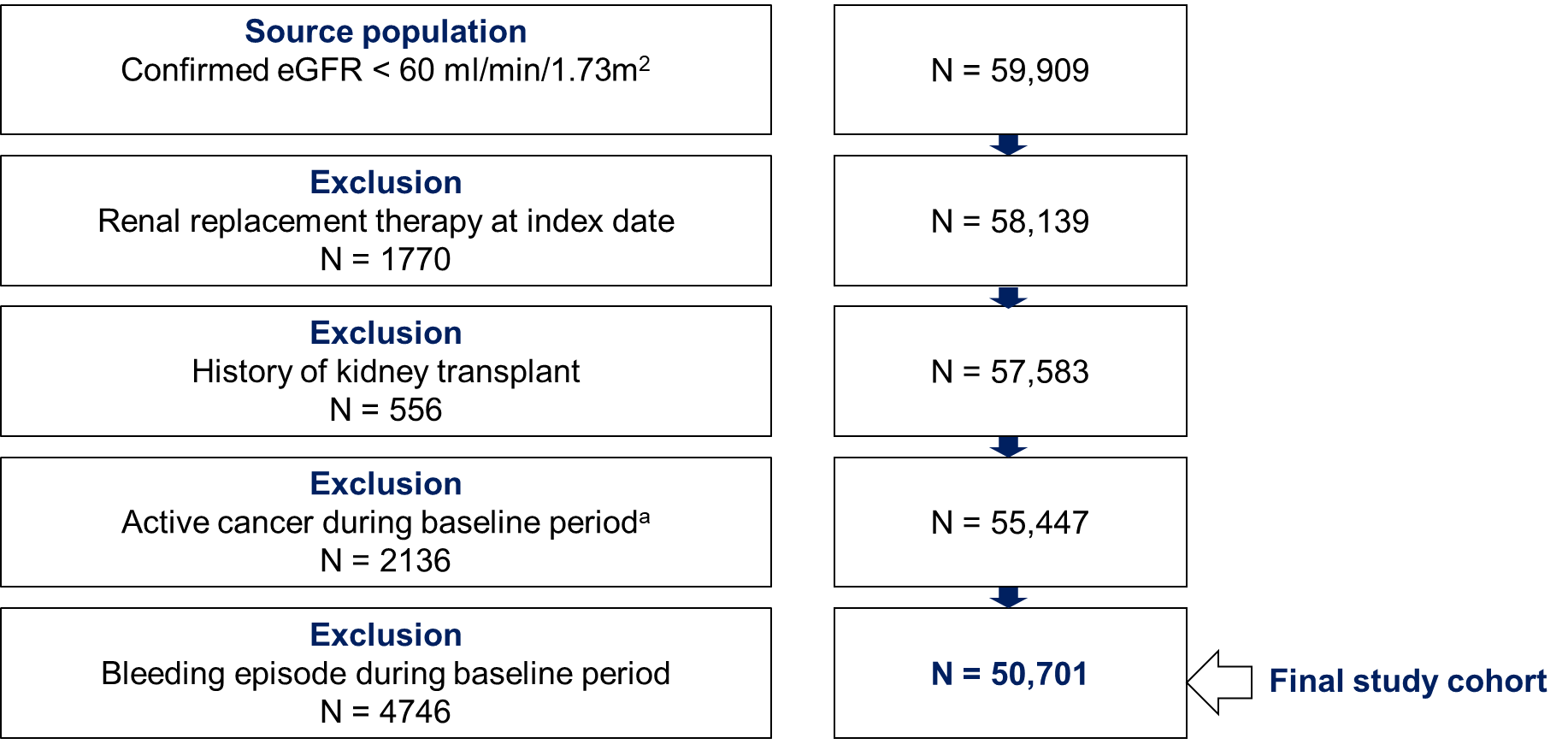


^a^Diagnosis codes (International Classification of Diseases, 9/10th revision [ICD 9/10]) and procedure codes (ICD 9/10 and Current Procedural Terminology, 4th Edition) for chemotherapy or radiation therapy were used to identify patients with active cancer.

*eGFR* estimated glomerular filtration rate.

Appendix Fig. S2 Kaplan–Meier curves for renal outcomes stratified by CKD stage over 5 years of follow-up, in patients without anemia (A) and patients with anemia at baseline (B)


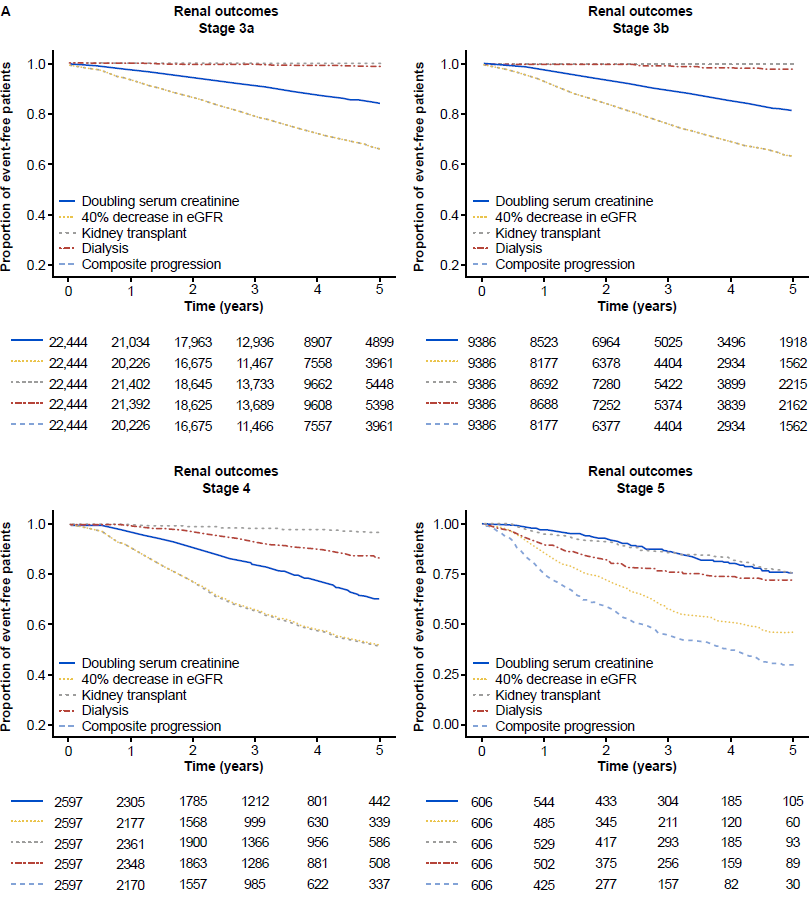


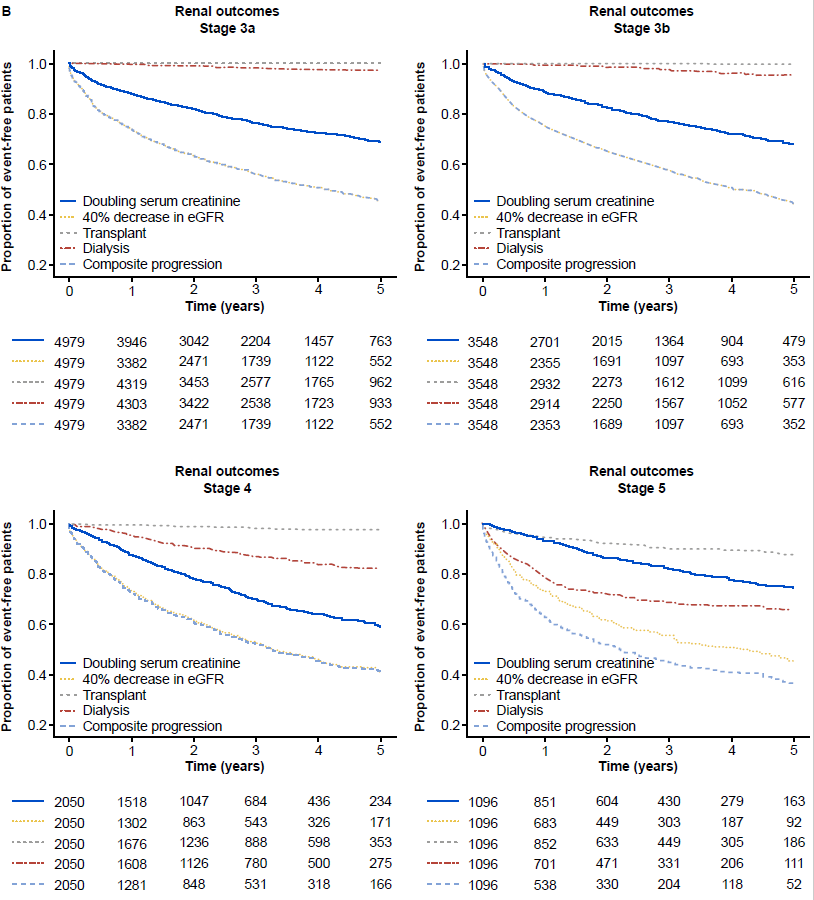


Baseline anemia was defined as Hb < 10 g/dL, determined from the lowest available Hb level within 6 months of the index date.

*CKD* chronic kidney disease, *eGFR* estimated glomerular filtration rate, *Hb* hemoglobin.

Appendix Fig. S3 Kaplan–Meier curves for cardiovascular outcomes stratified by CKD stage over 5 years of follow-up, in patients without anemia (A) and patients with anemia (B)


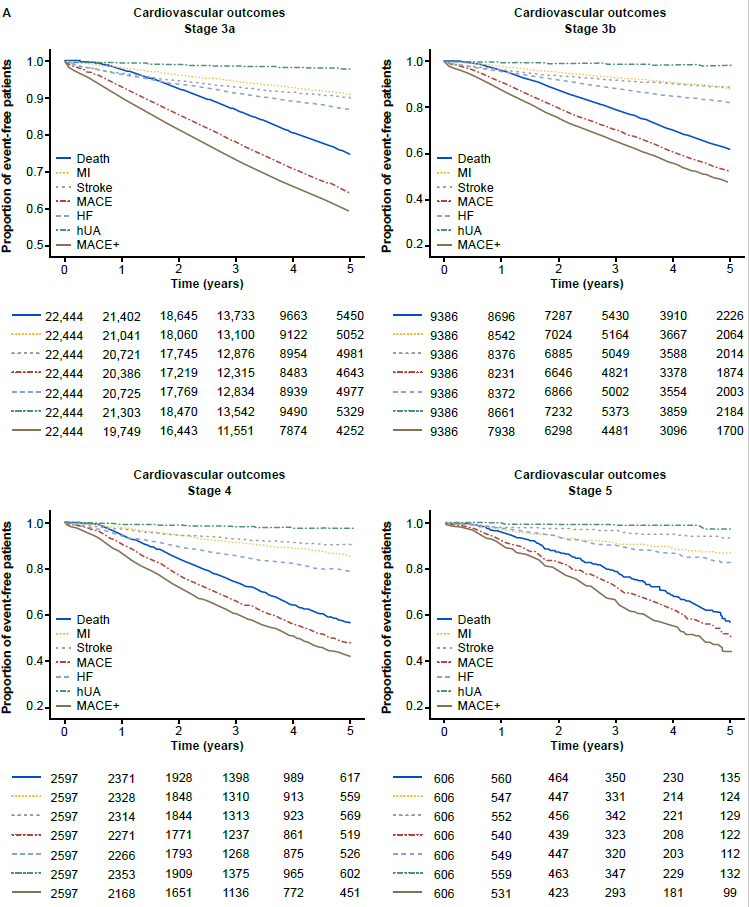


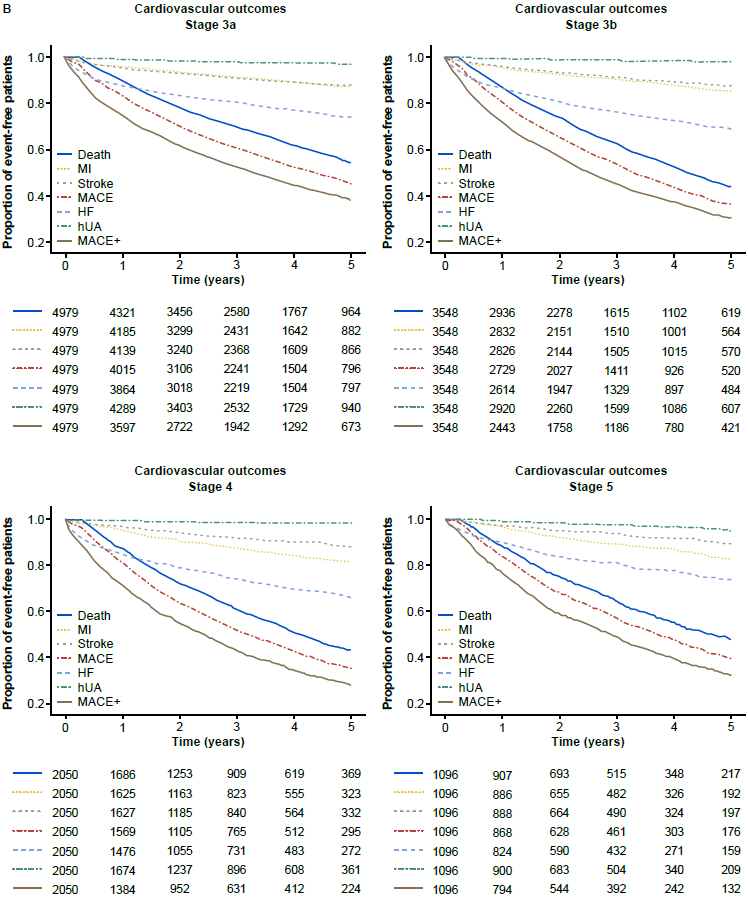


Baseline anemia was defined as Hb < 10 g/dL, determined from the lowest available Hb level within 6 months of the index date.

*Hb* hemoglobin, *HF* heart failure, *hHF* hospitalization for heart failure, *hUA* hospitalization for unstable angina, *MACE* major adverse cardiovascular events (first occurrence of the composite of all-cause mortality, non-fatal MI or non-fatal stroke), *MACE+* first occurrence of MACE outcome, hUA or hHF, *MI* myocardial infarction.

Appendix Fig. S4 Competing risk models for 5-year outcomes (CIF)

**Renal outcomes**


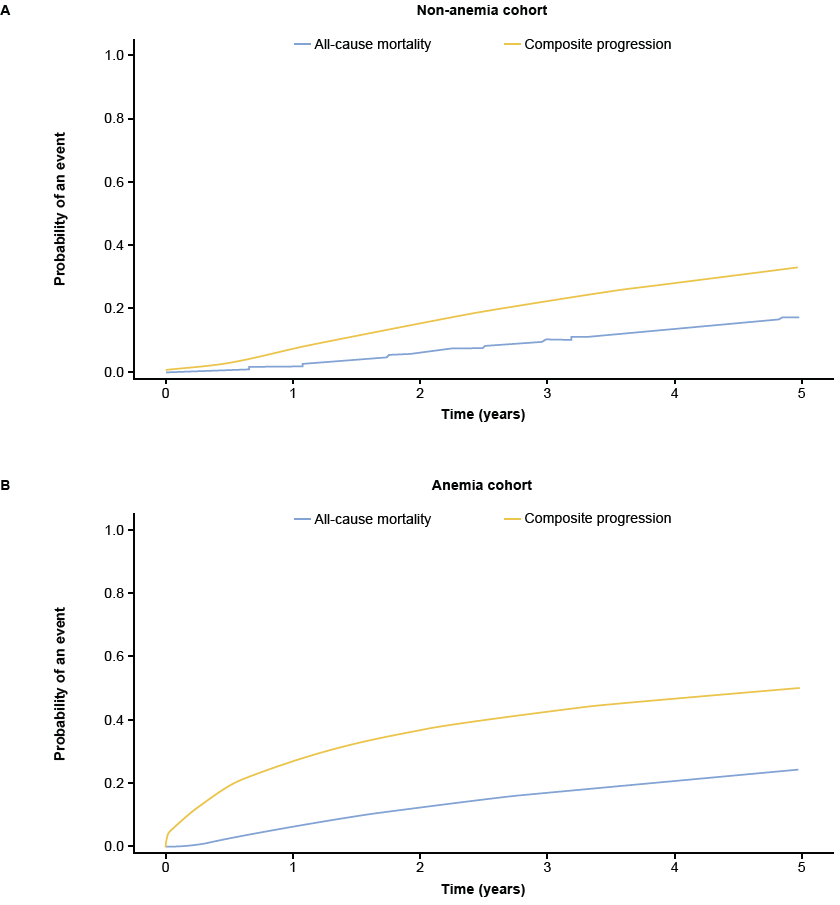


**Cardiovascular outcomes**


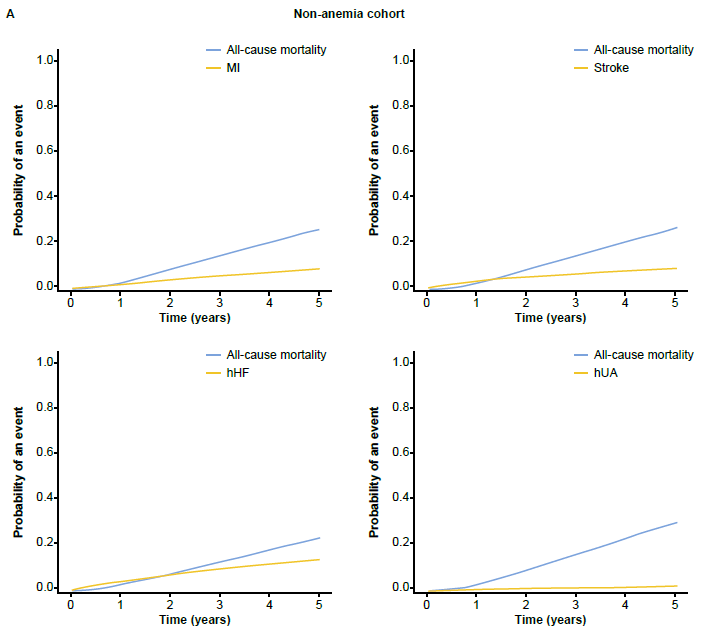


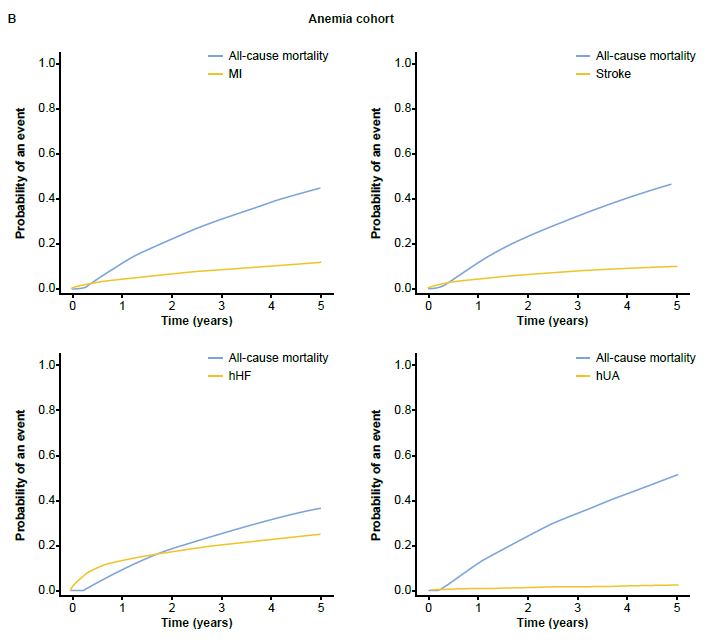


For all-cause mortality, per definition, the CIF and Kaplan–Meier curves are identical.

*CIF* cumulative incidence function, *hHF* hospitalization for heart failure, *hUA* hospitalization for unstable angina, *MI* myocardial infarction.

Appendix Fig. S5 Kaplan–Meier curves for bleeding and hospitalization outcomes in patients without anemia (A) and patients with anemia (B)


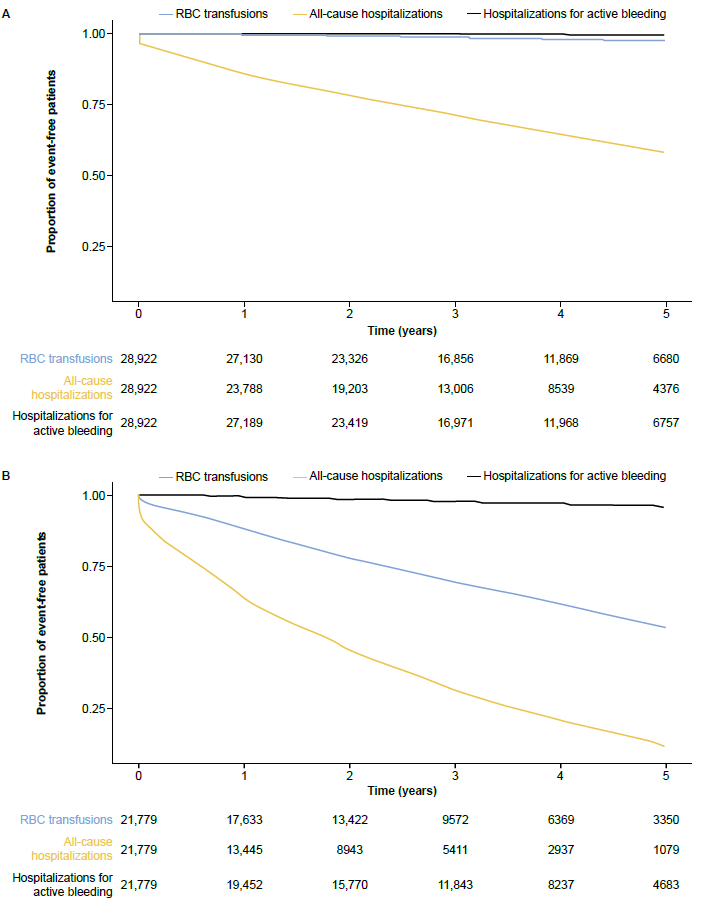


Baseline anemia was defined as Hb < 10 g/dL, determined from the lowest available Hb level within 6 months of the index date.

*Hb* hemoglobin, *RBC* red blood cell.

Appendix Fig. S6 Multivariable HRs for the association between baseline factors and incident anemia over 1 year and after 5 years of follow-up


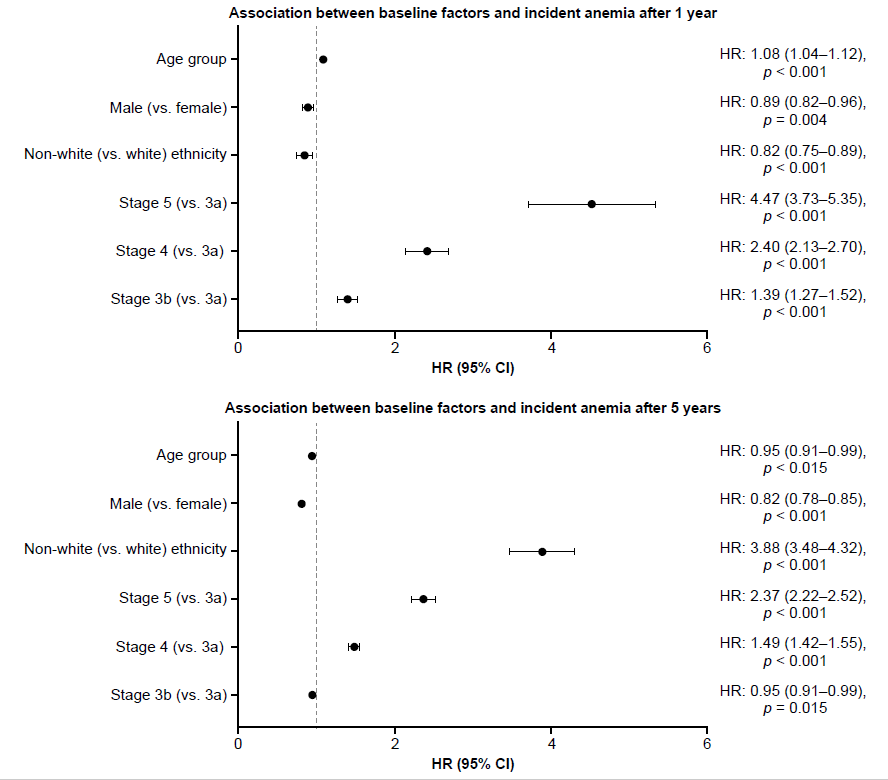


Multivariable HRs were calculated using Cox proportional hazards models, simultaneously adjusted for baseline covariates sex, age strata [<50; 50–59; 60–69; 70–79; and 80+ years], ethnicity, and CKD stage.

*CI* confidence interval, *CKD* chronic kidney disease, *HR* hazard ratio.
